# Uracil as a biomarker for spatial pyrimidine metabolism in the development of gingivobuccal oral squamous cell carcinoma

**DOI:** 10.1038/s41598-024-62434-z

**Published:** 2024-05-21

**Authors:** Soni Shaikh, Sangramjit Basu, Swarnendu Bag, Ankita Chatterjee, Sourav Datta, Devmalya Banerjee, Kapila Manikantan, Indu Arun, Pattatheyil Arun, Nidhan K. Biswas, Arindam Maitra, Deepak Kumar Mishra, Partha P. Majumder, Harsh Dhar, Geetashree Mukherjee

**Affiliations:** 1 Tata Medical Center, 14 MAR (E-W), New Town, Rajarhat, Kolkata, WB 700160 India; 2 Tata Consultancy Services (TCS), Kolkata, WB India; 3 Tata Translational Cancer Research Centre (TTCRC), 14 MAR (E-W), New Town, Rajarhat, Kolkata, WB 700160 India; 4 CSIR-Institute of Genomics and Integrative Biology (IGIB), Mall Road, New Delhi, 110007 India; 5 National Institute of Biomedical Genomics, P.O.: N.S.S., Kalyani, WB 741251 India; 6 Narayana Superspeciality Hospital, 120, 1, Andul Rd, Shibpur, Howrah, WB 711103 India; 7 Medica Superspecialty Hospital, 127, Eastern Metropolitan Bypass, Nitai Nagar, Mukundapur, Kolkata, WB 700099 India; 8 John C. Martin Centre for Liver Research and Innovations, Sitala East, IILDS, Hospital Road, Rajpur Sonarpur, Kolkata, WB 700150 India

**Keywords:** GB-OSCC, Nuclear magnetic resonance, P2Y6receptor, Tumor microenvironment, Uracil, Biomarkers, Oncology

## Abstract

No biomarker has yet been identified that allows accurate diagnosis and prognosis of oral cancers. In this study, we investigated the presence of key metabolites in oral cancer using proton nuclear magnetic resonance (NMR) spectroscopy to identify metabolic biomarkers of gingivobuccal oral squamous cell carcinoma (GB-OSCC). NMR spectroscopy revealed that uracil was expressed in 83.09% of tumor tissues and pyrimidine metabolism was active in GB-OSCC; these results correlated well with immunohistochemistry (IHC) and RNA sequencing data. Based on further gene and protein analyses, we proposed a pathway for the production of uracil in GB-OSCC tissues. Uridinetriphosphate (UTP) is hydrolyzed to uridine diphosphate (UDP) by CD39 in the tumor microenvironment (TME). We hypothesized that UDP enters the cell with the help of the UDP-specific P2Y6 receptor for further processing by ENTPD4/5 to produce uracil. As the ATP reserves diminish, the weakened immune cells in the TME utilize pyrimidine metabolism as fuel for antitumor activity, and the same mechanism is hijacked by the tumor cells to promote their survival. Correspondingly, the differential expression of ENTPD4 and ENTPD5 in immune and tumor cells, respectively, indicatedtheir involvement in disease progression. Furthermore, higher uracil levels were detected in patients with lymph node metastasis, indicating that metastatic potential is increased in the presence of uracil. The presence of uracil and/or expression patterns of intermediate molecules in purine and pyrimidine pathways, such asCD39, CD73, and P2Y6 receptors together with ENTPD4 and ENTPD5, hold promise as biomarker(s) for oral cancer diagnosis and prognosis.

## Introduction

Cancers of the head and neck, including the oral cavity, are the seventh most common cancer globally. At 12%, the incidence of oral squamous cell carcinoma (OSCC) is currently very high among males in India, with 40% of such cases occurring in the gingivobuccal region (GB-OSCC) ^1,2^. In fact, India has one of the highest incidences of GB-OSCC worldwide. Lymph node metastasis and loco-regional recurrence are the primary causes of treatment failure in advanced stages of the disease ^3^, with a 5-year survival rate of 5–15% ^4^.Nodal metastases are the most significant adverse prognostic factor of GB-OSCC survival^5^.

Metabolically active cancer cells require a continuous supply of energy to sustain proliferation ^6^. The rate of nucleotide metabolism is directly proportional to tumor cell proliferation. Such metabolic changes also occur in the immune system with the transition from the resting to the stimulated effector state ^7^. Adenosine triphosphate (ATP) produced in the mitochondria by the tricarboxylic acid (TCA) cycle plays an important role in cell metabolism and is, in fact, the only form of energy that can be used directly for different metabolic processes. Other forms of chemical energy require conversion to ATP before they can be used ^8^.

In cancer, metabolic reprogramming, redox homeostasis, and related signaling molecules mediate the crosstalk between tumor cells and immune cells, resulting in an increase in extracellular ATP (eATP) and adenosine (ADO) in the tumor microenvironment (TME). Extracellular ATP boosts antitumor immune responses, while ADO attenuates immune responses against tumors ^9^. Therefore, eATP and ADO are important for maintaining homeostasis in tumor immunity. Adenosine can be converted to its base, adenine, and then to adenine monophosphate (AMP), either directly, or indirectly through deamination to inosine before conversion to AMP ^9,10^.

Similar to the functioning of ATP/ADO, extracellular uridine triphosphate (eUTP) is enzymatically hydrolyzed to metabolic uridine diphosphate (eUDP). Free eUDP accumulates on the cell surface by binding to P2Y6 receptors present in cell membrane. The sequential enzymatic degradation of UDP within the cell results in the formation of uracil. Although uracil is typically an RNA nucleotide, it is also found infrequently in DNA ^11^. Recent research has shown that adenosine turnover in the TME depends on the activity of ectonucleoside triphosphate diphosphohydrolase1(CD39)/5'-nucleotidase ecto (CD73) in oral squamous cell carcinoma ^12^.We have also observed high levels of purine metabolism in oral cancer ^13^. Others have observed the enrichment of CD39 in the TME, which hydrolyzes NTPs (ATP, UTP, etc.) preferentially over NDPs (ADP, UDP, etc.). Intracellular NTPDase-4 and NTPDase-5 hydrolyze UDP preferentially over the other NDPs ^11,14^, resulting in the formation of uracil.

In view of the alterations in metabolism with disease progression, a comprehensive understanding of the metabolic heterogeneity and plasticity that exist between the tumor and its microenvironment is required to identify novel targets for therapeutic approaches. Therefore, in this study, we aimed to identify metabolic biomarkers of GB-OSCC through NMR-based metabolomics analysis combined with RNA-sequencing and immunohistochemistry investigations.

## Materials and methods

### Patients and sample collection

The following inclusion criteria were applied: Patients aged > 18 years; with newly diagnosed oral gingivobuccal squamous carcinomas defined as T1 to T4 according to the TNM Classification of the American Joint Committee on Cancer (8th Edition); eligible to receive institutional standard care for the disease and periodic follow-up thereafter; consented to participation in the study. Patients who have received prior treatment (chemotherapy/radiotherapy) were excluded. The characteristics of eligible GB-OSCC patients are presented in Table 1.

**Table 1:** Patient details. Characteristics of patients whose samples were used for NMR (n = 71), RNA-Seq (n = 46), and IHC (n = 46). Samples in the table are according to age, sex, node status, perineural invasion (PNI), and tumor grade. Y = yes; N = no.

Serial no	Age	Sex	Grade	Size (Maximum dimension) cm	PNI	No. of nodes	Tobacco use	Experiments
1	34	M	3	2.6	Y	0	Y	NMR + IHC + RNAseq
2	39	M	2	6	N	0	Y	NMR
3	42	M	2	2.5	Y	0	Y	NMR + IHC + RNAseq
4	42	M	1	5.3	N	0	Y	NMR
5	43	M	1	1	N	0	Y	NMR + IHC + RNAseq
6	43	M	2	2.8	Y	0	Y	NMR + IHC + RNAseq
7	44	M	2	3	N	0	Y	NMR + IHC + RNAseq
8	44	M	2	1.9	Y	0	Y	NMR
9	44	M	1	1.6	N	0	Y	NMR
10	44	M	2	3	N	0	Y	NMR
11	45	M	2	5	Y	0	Y	NMR
12	45	M	2	2.8	N	0	Y	NMR
13	46	M	2	3	N	0	Y	NMR
14	47	M	3	2.6	N	0	Y	NMR + IHC + RNAseq
15	47	M	3	3	N	0	Y	NMR
16	50	F	2	3.1	N	0	Y	NMR
17	52	F	2	0.7	N	0	Y	NMR + IHC + RNAseq
18	52	F	2	3.4	N	0	Y	NMR + IHC + RNAseq
19	52	F	3	2.2	Y	0	Y	NMR
20	52	M	2	2	N	0	Y	NMR
21	55	M	2	2.5	Y	0	Y	NMR + IHC + RNAseq
22	56	M	1	8	N	0	Y	NMR + IHC + RNAseq
23	57	M	2	2.2	N	0	Y	NMR + IHC + RNAseq
24	58	M	2	6	N	0	Y	NMR + IHC + RNAseq
25	58	M	2	3	N	0	Y	NMR + IHC + RNAseq
26	58	M	2	10	N	0	Y	NMR + IHC + RNAseq
27	58	F	2	5	N	0	Y	NMR
28	58	M	2	10	N	0	Y	NMR
29	59	M	1	3.5	N	0	Y	NMR + IHC + RNAseq
30	59	M	2	3.5	N	0	Y	NMR + IHC + RNAseq
31	60	F	1	2.3	N	0	Y	NMR
32	60	F	3	2.8	N	0	Y	NMR
33	61	M	2	6.2	N	0	Y	NMR
34	61	M	1	˂0.1	N	0	Y	NMR
35	61	F	2	3.5	N	0	Y	NMR
36	62	M	3	2.2	Y	0	Y	NMR + IHC + RNAseq
37	63	M	2	1	N	0	Y	NMR
38	66	M	1	3.7	N	0	Y	NMR
39	66	M	2	1	N	0	Y	NMR
40	67	M	2	2.6	N	0	Y	NMR + IHC + RNAseq
41	68	M	2	11	N	0	Y	NMR + IHC + RNAseq
42	68	F	3	2.6	N	0	Y	NMR
43	68	M	2	11	N	0	Y	NMR
44	68	F	3	2.6	N	0	Y	NMR + IHC + RNAseq
45	71	M	1	2.5	N	0	Y	NMR
46	72	M	3	1.8	N	0	Y	NMR + IHC + RNAseq
47	75	M	2	2.5	Y	0	Y	NMR + IHC + RNAseq
48	75	M	2	3	N	0	Y	NMR + IHC + RNAseq
49	50	M	2	5.3	Y	1	Y	NMR + IHC + RNAseq
50	53	M	2	3.3	N	1	Y	NMR + IHC + RNAseq
51	57	M	3	3.5	Y	1	Y	NMR + IHC + RNAseq
52	60	M	2	2.5	N	1	Y	NMR + IHC + RNAseq
53	62	M	2	3.9	N	1	Y	NMR + IHC + RNAseq
54	64	M	2	3.6	N	1	Y	NMR + IHC + RNAseq
55	64	M	3	1.6	N	1	Y	NMR + IHC + RNAseq
56	71	F	2	3.2	Y	1	Y	NMR + IHC + RNAseq
57	72	F	3	3.2	N	1	Y	NMR + IHC + RNAseq
58	75	F	1	2.2	N	1	Y	NMR + IHC + RNAseq
59	61	M	2	4.8	Y	2	Y	NMR + IHC + RNAseq
60	65	M	2	5.2	Y	2	Y	NMR + IHC + RNAseq
61	65	M	1	5.1	N	2	Y	NMR + IHC + RNAseq
62	69	M	2	2.7	N	2	Y	NMR + IHC + RNAseq
63	45	M	2	3.5	Y	3	Y	NMR + IHC + RNAseq
64	49	M	3	5.2	Y	3	Y	NMR + IHC + RNAseq
65	71	M	3	3.2	Y	3	Y	NMR + IHC + RNAseq
66	35	M	2	5.5	Y	4	Y	NMR + IHC + RNAseq
67	59	F	3	4.3	N	4	Y	NMR + IHC + RNAseq
68	57	M	3	3.6	Y	5	Y	NMR + IHC + RNAseq
69	61	F	1	3.5	N	5	Y	NMR + IHC + RNAseq
70	64	F	2	3.5	N	6	Y	NMR + IHC + RNAseq
71	54	M	3	2.5	Y	21	Y	NMR + IHC + RNAseq

Tumor tissues were collected from specimens of GB-OSCC obtained during primary surgical resection (written informed consent was obtained from all subjects); tumor-adjacent (approximately 5 cm) normal tissues were also obtained. Samples were immediately snap-frozen in liquid nitrogen and stored at -80 °C. Formalin-fixed paraffin-embedded (FFPE) tissues were used for immunohistochemistry. The GBC035 cell culture model of buccal mucosal oral cancer was developed and provided by Singh *et. al*. (National Institute of Biomedical Genomics, India) ^15^. The study was approved by the Tata Medical Center Institutional Review Board (IRB) (Ref. no. EC/GOVT/23/17) and was conducted in accordance with the Declaration of Helsinki.

### NMR

Tumor samples from 71 patients, 25 normal tissue samples (used as batch controls), and 10 passages of cell lines of GB-OSCC were used in the NMR study. Metabolite extraction was performed according to a previously reported method (Supplementary Data 1)^16^. Subsequently, metabolites were extracted from the supernatants with 80% methanol and dried using a vacuum centrifuge (Concentrator Plus, Eppendorf) without heat. Each sample was then reconstituted in 600 μl PBS buffer prepared in D_2_O (pH7.4) with trimethylsilylpropanoic acid (TSP) as the internal reference. The NMR spectra of the samples were obtained using a Bruker Avance III HD 700. The water-suppressed proton NMR peaks were analyzed with TopSpin4.1.1 software (Bruker) (https://www.bruker.com/en/products-and-solutions/mr/nmr-software/topspin.html) and metabolites were identified by searching the Biological Magnetic Resonance Data Bank (BMRB) database.

### Immunohistochemistry (IHC) staining and scoring

Tissue microarrays (TMAs; diameter 0.6 mm) were constructed from FFPE samples (46 of 71 cases) using a Beecher Instruments automated tissue arrayer (Estigen Tissue Science; https://www.estigen.com) according to the manufacturer’s instructions^17^. Four TMAs were prepared from each tumor; two from the invasive margins and two from the center ^18^; a few normal squamous epithelium and stroma tissue samples were included as controls. Sections (thickness 5 μm) were prepared from the array blocks and dried at 60 °C for 30 min. IHC was performed in a Bond Max Automated Immuno-histochemistry Vision Bio-system (Leica Microsystems GmbH, Wetzlar, Germany) using standard protocols ^13^. The selected markers were detected using the following antibodies: CD39 (Abcam Cat. No. ab223842, clone EPR20627; rabbit monoclonal; 1:500 dilution; control—human colon carcinoma), CD73 (Abcam Cat. No. ab133582, clone EPR6114; rabbit monoclonal; 1:100 dilution; control—human tonsil tissue), P2Y6 receptor (Abcam Cat. No. ab92504, clone EPR3816; rabbit monoclonal; 1:200 dilution; control– human tonsil tissue), ENTPD4 (Sigma-Aldrich Cat. No. HPA017655, rabbit polyclonal; 1:100 dilution; control—human kidney tissue) and ENTPD5 (Abcam Cat. No. ab108603, clone: EPR3784; rabbit monoclonal; 1:50 dilution; control—human kidney tissue). The method used for antibody validation is available in Supplementary Data 2.

Digital images of the stained slides were captured using the Aperio Versa 8 platform (Leica, Wetzlar Germany). Images were captured at 20 × magnification and analyzed using QuPath software, Version 0.1.2 (https://qupath.github.io/) (see Supplementary Data 3 for details). Marker expression was analyzed in normal epithelium, tumor cells, immune cells, and stromal cells both at the tumor margins and the tumor centre. The level of expression was measured by two independent analysts as the percentage of cells with a minimal intensity that was considered positive. In 7% of cases for which scoring seemed to be inaccurate due to mild background staining, the slides were assessed manually by two independent pathologists and the average score was used.

### RNA isolation and sequencing

RNA sequencing was performed on tumor and tumor-adjacent normal tissue samples collected from a subset of GB-OSCC patients recruited in this study (46 of 71 cases). For each tissue sample, the total RNA was extracted using the RNeasy kit (QIAGEN), and the RNA quality was assessed using Agilent 2100 Bioanalyzer and Nano Drop 2000 (Thermo Fisher Scientific). All isolated RNA samples were of high quality(OD260/OD280 ratio ≥ 2 and RNA integrity number (RIN) ≥ 7). RNA sequencing libraries were generated using TruSeq RNA Library Prep Kit v2 (Illumina) according to the manufacturer’s protocol. The final libraries were sequenced at a target of 100 million reads per sample on the sequencer (Illumina) using 2 × 100 cycles.

### RNA-seq data retrieval and analyses

Raw RNA-Seq sequencing data were converted to FASTQ files, and the read quality was determined using FASTQC (https://qubeshub.org/resources/fastqc). Low-quality reads and adapter sequences were removed. High-quality reads were mapped to the human genome assembly hg19 (NCBI 37) reference transcriptome using Bowtie 2 with the no-discordant option to use paired reads originating from actual single fragments. The results were acquired in BAM format. After sorting indexes using Samtools, the resulting BAM files were used to extract transcript read counts using Salmon. Gene-level TPM counts were generated using Tximport. Genes with < 1 transcripts per million (TPM)in ≥ 80% of the samples were filtered from the downstream analysis. Data were then converted to zero median values with unit standard deviation (SD) to construct the matrix in a form suitable for downstream analyses. The differential gene expression between normal and tumor samples was calculated using DESeq2. Gene set enrichment analysis was performed using the GSEA package to identify the reactome pathways, canonical Kyoto the Encyclopedia of Genes and Genomes(KEGG) pathways, and WikiPathways from MolSigDB. Other enrichment analyses of characteristics, such as gene ontology (GO) and transcription factor binding motifs, were performed using EnrichR (web version). Data plotting and exploratory data analyses were performed using the Tidyverse and ggpubr packages in R.

### Statistical analysis

Raw experimental data were converted into a frequency domain spectrum by discrete Fourier transformation. Subsequently, the statistically significant (*P* < 0.05) peaks were subjected to spectral integration using MestReNova software (Mestrelab Research Mnova 7.1.0) (https://mestrelab.com/download_file/mnova-7-1-0/ ). Baseline corrections were carried out wherever necessary. Non-parametric Mann–Whitney U-tests were used to identify the statistically significant differential metabolite peaks between tumor and normal tissue. Statistical analysis was performed using R (R i386, version 3.6.2) and SPSS software *(IBM SPSS Statistics)* (version 20). https://www.ibm.com/products/spss-statistics?utm_content=SRCWW&p1=Search&p4=43700078595923344&p5=p&gad_source=1&gclid=Cj0KCQiA5rGuBhCnARIsAN11vgR4qTbiZlKnrXOJkB25YR409m7HCe27nENgiSX4WaIqTC1LH__2OvkaAs5pEALw_wcB&gclsrc=aw.ds.

Metabolite identification and pathway enrichment analyses were performed with MetaboAnalyst 5.0 software (https://www.metaboanalyst.ca/) linked to the Human Metabolome and KEGG databases. Data were log-transformed (base 10) before pathway enrichment analysis (Supplementary Data 4). IHC scores were represented as mean ± SD and analyzed by paired Student’s *t*-test. *P* ≤ 0.05 was considered to indicate statistical significance.

### Ethical approval

The project was approved by the Tata Medical Center Institutional Review Board (IRB) (Ref. no. EC/GOVT/23/17).

## Results

### Patient details

In this study, GB-OSCC was found to be a male-dominant disease with the majority of patients aged > 50 years. All cases were diagnosed as squamous cell carcinoma, and the majority were moderately differentiated tumors. Lymph node metastases were detected at diagnosis in 23 cases (Table 1).

### Identification of NMR peaks characteristic of uracil

**Figure 1:**
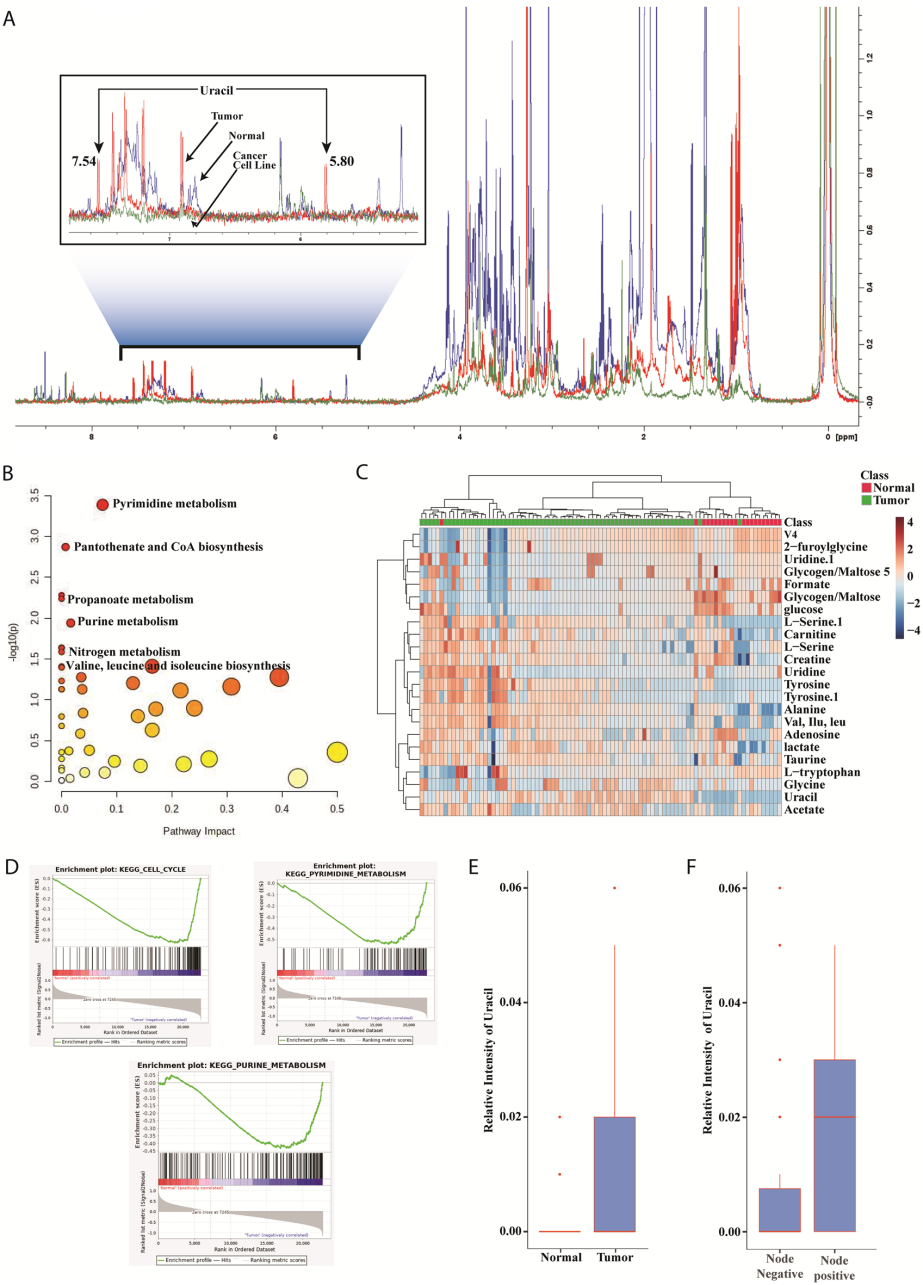
NMR studies. (**A**) Normal (n = 25) vs. GB-OSCC tumor tissue (n = 71) and cell line (n = 10). Merged visualization of NMR peaks in which the red spectrum represents the tumor sample, green represents the cell line sample, and the blue spectrum represents normal tissue. The characteristic peaks for uracil appeared at δ5.80 and δ7.54 for the GB-OSCC samples, but were absent from the cell samples and normal tissues. (**B**) Pathway studies revealed that pyrimidine and purine metabolism were significantly upregulated in GB-OSCC cells compared to normal cells (*P* = 4e^-4^for pyrimidine metabolism and *P* = 1.15e^-2^ for purine metabolism). (**C**) Heat map ofthe expression intensity of metabolites in the samples showing intense expression of uracil in cancer cells compared to normal cells. (**D**) Pathway studies of RNA-seq data showing prominent energy signaling consistent with the NMR findings. (E, F) Uracil expression was higher in the tumor samples compared to the normal samples (*P* = 1.79439e^-4^), and was significantly upregulated in node-positive cases (*P* = 4.83826e^-4^). Node-negative (n = 48) vs. node-positive (n = 23).

Several metabolites, including key energy metabolites, were identified by NMR analysis. Among the metabolites detected, uracil was prominently expressed in tumor tissues (Red spectrum), with peaks at δ5.80 and δ7.54 that were consistent with the H9 and H10 δ-shifts of 5.7915 and 7.5261, respectively, for uracil in the BMRB database (bmse000187), indicating the presence of uracil in 83.09% of the tumor samples. These characteristic peaks were not detected in the normal tissue samples (Blue spectrum) or primary GB-OSCC cell lines (Green spectrum) (Fig. 1A). Additional peaks upfield and/or downfield of δ5.80 and δ7.54, causing mismatches with the specific NMR spectrum, were not considered.

## Pathway impact of NMR peaks and correlation of uracil with lymph node metastasis

Compared to normal tissues, pyrimidine and purine metabolism were significantly upregulated in GB-OSCC tissues (*P* = 0.0004; log10(*P*) = −3.3859 for pyrimidine metabolism and *P* = 0.0115; log10(*P*) = −1.9402 for purine metabolism) (Fig. 1B). Furthermore, uracil was highly upregulated in cancer tissues compared to normal tissues (Fig. 1C). In addition to nucleotide metabolism, significant (*P* < 0.05) upregulation of pantothenate and CoA biosynthesis, propanoate metabolism, and nitrogen metabolism as well as valine, leucine, and isoleucine biosynthesis was detected (log10(*P*)values:-2.87, -2.237, -1.6336 and-1.4078, respectively) (Fig. 1B). Enrichment of the energy metabolism pathways was also supported by the RNA-Seq data (Fig. 1D). In addition to the overproduction of uracil in tumor tissues, uracil expression was also significantly upregulated (*P* < 0.05) in the lymph node metastasis-positive (node-positive) cases compared to the lymph node metastasis-negative (node-negative) cases (Fig. 1E,F), indicating that uracil expression also correlates with lymph node status.

### Necrosis in the TME

**Figure 2:**
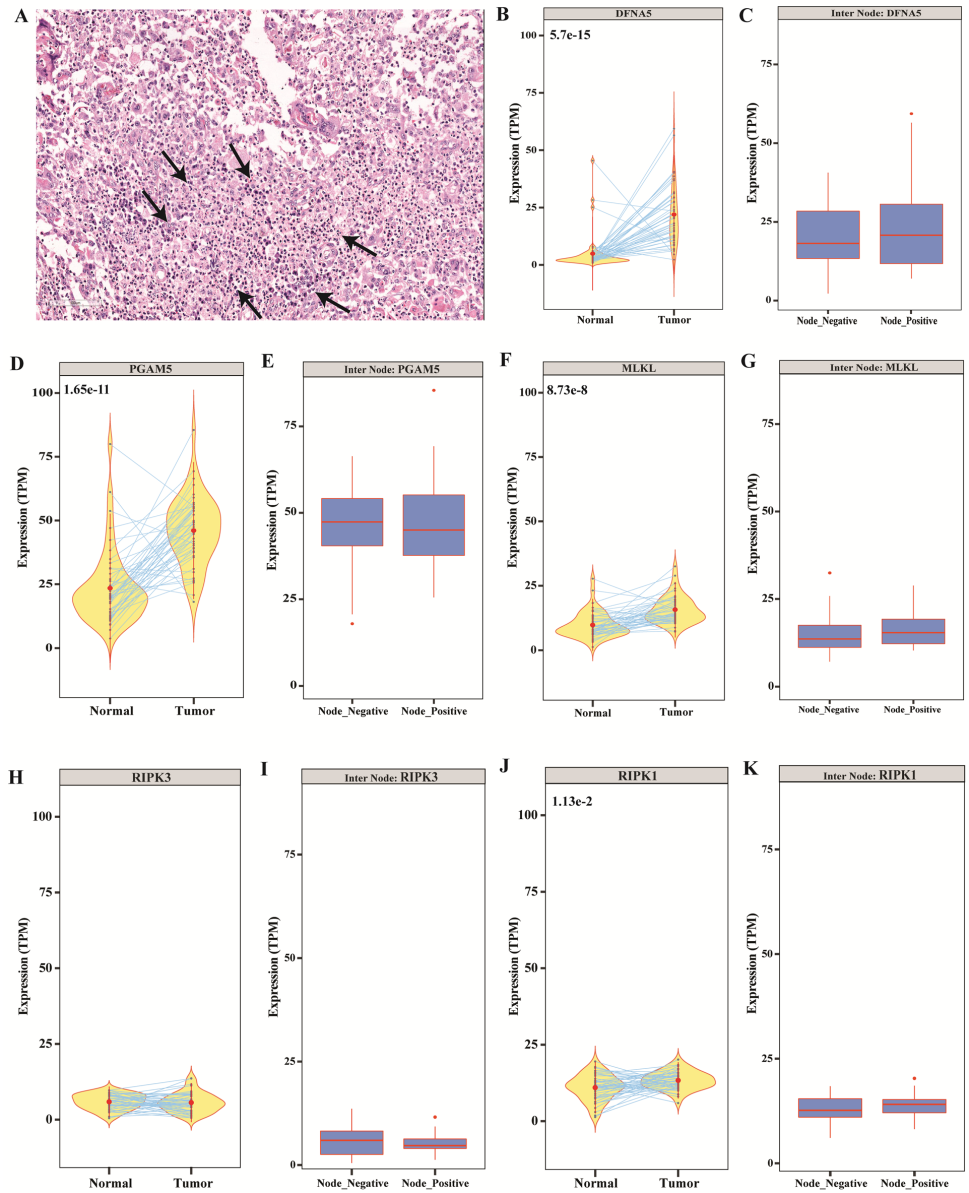
Necrosis evaluation. (**A**) H&E-stained sections showing necrotic features (arrows) in the stroma of GB-OSCC.(**B–K**) Four pro-necrotic genes *DFNA5* (*P* = 5.7e^-5^; n = 46), *PGAM5* (*P* = 1.65e^-11^; n = 46), *MLKL* (*P* = 8.73e^-8^; n = 46)and *RIPK1* (*P* = 1.13e^-2^; n = 46)were differentially expressed in tumor cases, while *RIPK3* was not(not significant; n = 46). However, there was no significant difference in the expression of the five genes when node-positive cases (n = 23) were compared with node-negative cases (n = 23).

Accidental cell death, known as necrosis, is not generally subject to cellular regulation and is characterized morphologically by increased cell volume and swelling of organelles, plasma membrane rupture, and leakage of intracellular molecules, including metabolites ^19^. Hematoxylin and eosin (H&E) staining of GB-OSCC tissue sections exhibited features of necrosis (Fig. 2A). Furthermore, expression of pro-necrotic genes, such as *PGAM5* and *DFNA5*^20^, were significantly upregulated in tumor samples compared to normal samples. In addition, upregulated *DFNA5* expression correlated with the node-positive status, while upregulated *PGAM5* correlated with node-negative status (Fig. 2B–K).

## CD39, CD73, and P2Y6receptor expression patterns in the TME and correlation with lymph node status

**Figure 3:**
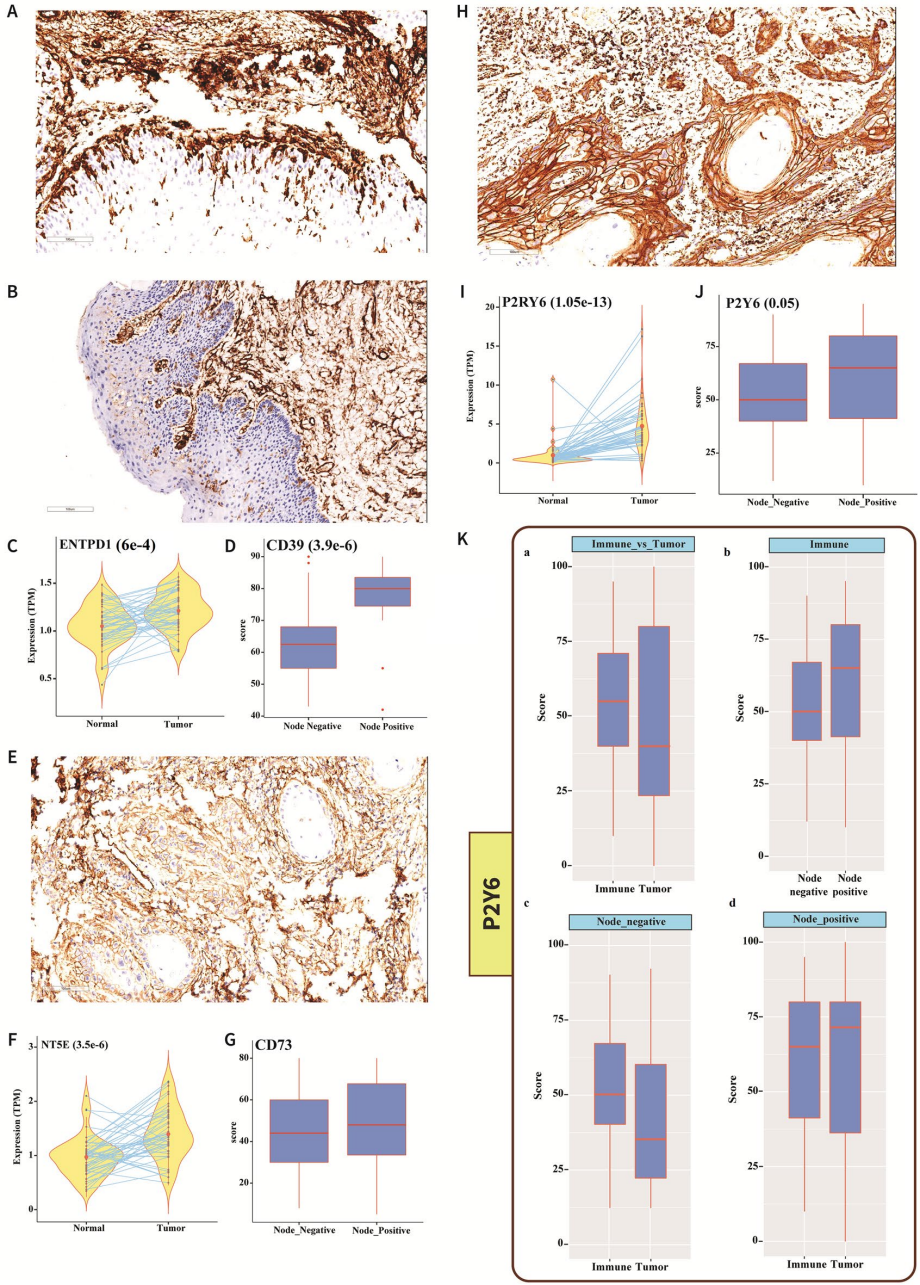
Expression patterns of CD39, CD73, and P2Y6 receptor. (**A**, **B**) CD39 was significantly upregulated in the TME. Neither the tumor cells nor the normal epithelium was CD39^+^ according to IHC. (**C**) RNA-Seq data demonstrated significant upregulation of the CD39-encoding gene, *ENTPD1*, in tumors (*P* = 6e^-4^; n = 46). (**D**) IHC scoring confirmed CD39 enrichment in node-positive cases (*P* = 3.9e^-6^; n = 23). (**E**) IHC studies demonstrated CD73 over expression in the tumor stroma, while CD73 was not expressed in the tumor region. (**F**) RNA-Seq data showing expression of the gene encoding CD73, *NT5E*, solely in the stroma (*P* = 3.5e^-6^; n = 46). (**G**) CD73 score did not differ significantly according to the node status (n = 23). (**H**) The GPCR membrane protein, P2Y6 receptor was widely distributed within the stroma and was prominent in the invasive tumor margins, whereas distant tumor cells were P2Y6 receptor-negative. (**I**) TheP2Y6 receptor gene, *P2RY6,*was upregulated in tumor samples (*P* = 1.05e^-13^; n = 46). (**J**) A proportional increase in P2Y6 receptor protein was observed in node-positive cases (*P* = 0.05; n = 23). (**K**) (**a**) P2Y6 receptor was expressed at higher levels in immune cells compared with tumor cells. (**b**) Immune cells from node-positive cases showed higher P2Y6 receptor expression than node-negative immune cells. (**c**) In node-negative cases, P2Y6 receptor was more widely distributed in immune cells, but more widely distributed in tumor cells in node positive-cases.

We analyzed the expression patterns of CD39, CD73, and P2Y6 receptors using IHC. CD39 was significantly over expressed in the tumor stroma, but was not detected in tumor cells and normal epithelium (Fig. 3A,B). This finding was further supported by our RNA-Seq data showing over expression (twofold) of the CD39-encoding gene, *ENTPD1*,in tumor samples compared to normal samples (Fig. 3C). CD39 upregulation was also positively correlated with lymph node metastasis (Fig. 3D). Similarly, CD73 was also significantly over expressed in the TME, and the corresponding gene*, NT5E*, was upregulated (fivefold) compared to normal samples (Fig. 3E,F). Interestingly, CD73 was not significantly expressed in samples from node-positive patients compared to those from node-negative patients (Fig. 3G). Expression of the P2Y6 receptor, a G protein-coupled receptor (GPCR) membrane protein, showed variable distribution within the stroma, with intense staining in the tumor-infiltrating immune cells, where it was localized in the membrane and cytosol. Tumor cells showed P2Y6 receptor membrane-positivity and cells adjacent to the immune cells at the invasive margins showed cytoplasmic positivity, whereas cells that were distant from the immune cells were negative or showed weak membrane-positivity (Fig. 3H). The high expression of the P2Y6 receptor protein detected by IHC supported the corresponding upregulation (12-fold) of the *P2RY6* gene compared with the normal samples (Fig. 3I). The total P2Y6 receptor protein expression correlated positively with node-positive cases (Fig. 3J). In addition, expression of the P2Y6 receptor in immune cells was high and correlated with lymph node metastasis (Figs. 3Ka, 4Kb). Separate analysis of the protein levels in immune cells and tumor cells showed significantly increased P2Y6 receptor staining in immune cells from node-negative cases (Fig. 3Kc), while in node-positive cases, P2Y6 receptor expression was higher in tumor cells than in immune cells (Fig. 3Kd). The significant difference in the protein expression levels between immune and tumor cells in node-negative cases indicates pre-activation of the P2Y6 receptor in the immune cells. Based on our findings, we hypothesized that with disease progression (node positivity), tumor cells in proximity to immune cells adapt this mechanism and utilize the P2Y6 receptor to gain more energy, thus accounting for the positive correlation between the P2Y6 receptor expression in tumor cells and in nodal metastasis.

**Figure 4:**
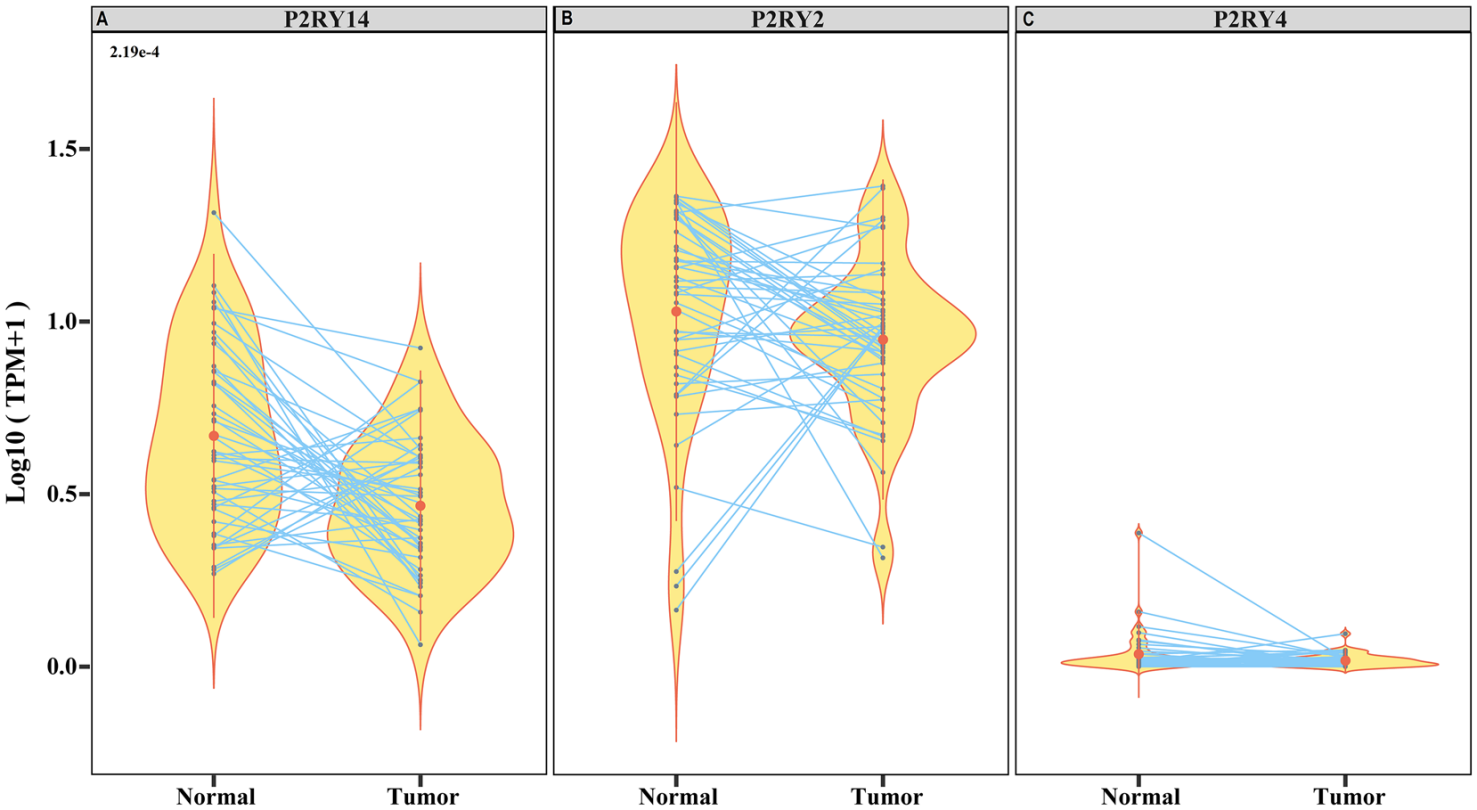
Expression of other UDP-specific P2Y-receptor subtypes. In addition to P2RY6, other UDP-specific genes, namely (**A**) *P2RY14 (P* = 2.19e^-4^*;* n = 46) was significantly over expressed in normal tissue. (**B**) *P2RY2*, and (**C**) *P2RY4*,were expressed at levels that were insufficient to participate in UDP accumulation.

### UDP accumulation by P2Y-receptors

To date, eight genes encoding P2Yreceptors have been cloned in humans. The G-protein-coupled P2Y1-like receptors include P2Y1 (ligand ADP), P2Y2 (ligands UTP, ATP), P2Y4 (ligands UTP, UDP, GTP, ATP), P2Y6 (ligand UDP), and P2Y11 (ligand ATP). The G-protein-coupled P2Y12-like receptors are P2Y12 (ligand ADP), P2Y13 (ligand ADP), and P2Y14 (ligands UDP, UDP-glucose) ^21^. In this study, the significant over expression of uracil in tumor tissues (Fig. 1A) correlated with the gene expression of P2Y-receptors with an affinity with uracil precursor molecules (UTP, UDP, UDP-glucose), including *P2RY2, P2RY4, P2RY6,* and *P2RY14*. Specifically, we detected upregulated (12-fold) expression of the UDP-specific *P2RY6* gene in tumor samples compared with normal samples (Fig. 3I). Quantification of the other subtypes confirmed that *P2RY2, P2RY4* and *P2RY14* were not significantly upregulated in the tumor samples compared to normal samples (Fig. 4A–C).

### UDP endocytosis and uracil production

**Figure 5:**
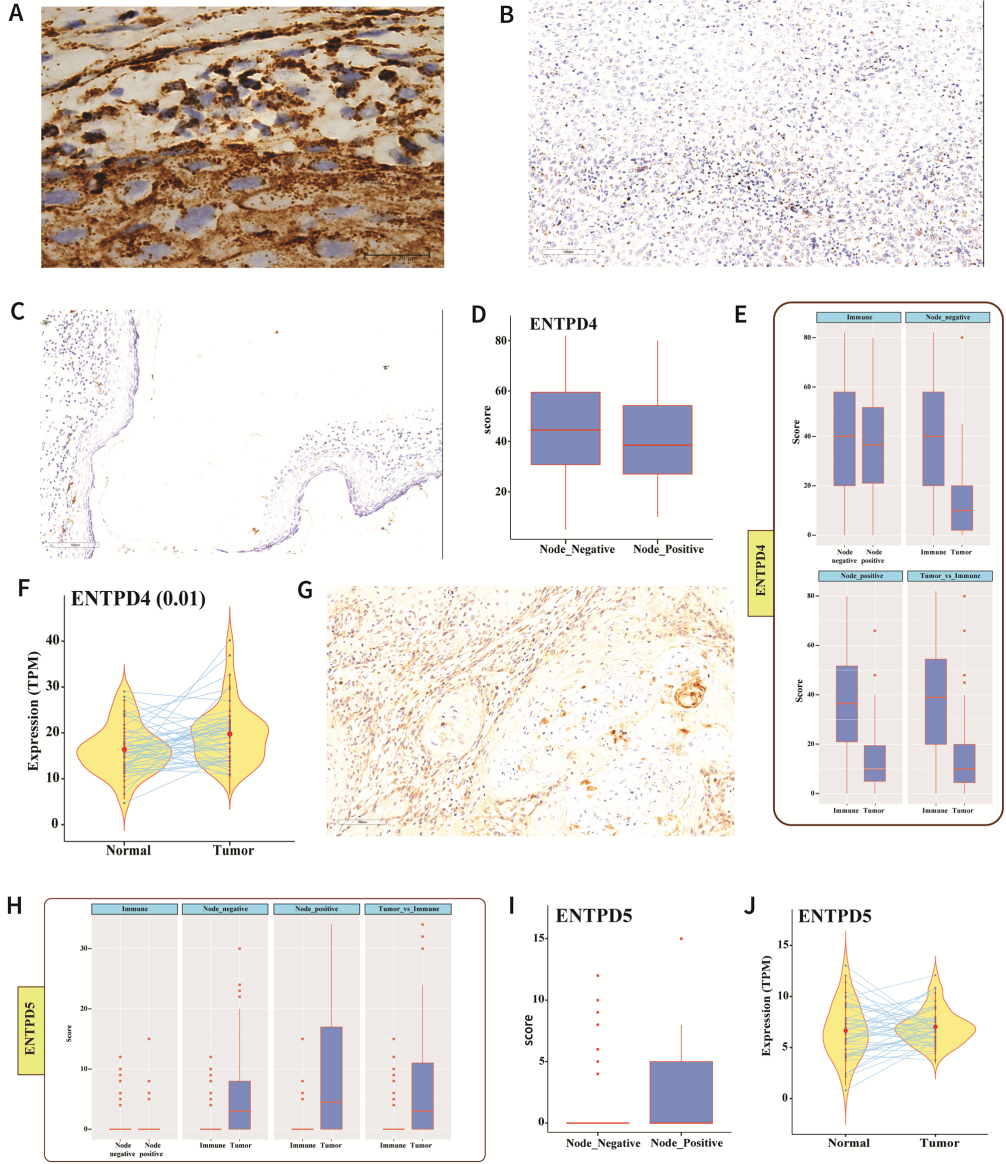
UDP endocytosis and UMP production. **(A)** IHC staining showing cytoplasmic expression of P2Y6 receptor both in the tumor and stroma (mainly at the invasive tumor margins);100 × magnification. (**B**, **C**) IHC staining showing over expression of the UDP-specific ENTPD4 in the stroma compared to the tumor region, and significant expression of ENTPD4 in the tumor compared to the normal epithelium. (**D**) Node-negative cases showed higher expression of ENTPD4 than node-positive cases. (**E**) Immune cells expressed significantly higher levels of ENTPD4 compared to tumor cells, while ENTPD4 expression was consistent in tumor cells, irrespective of nodal status. (**F**) RNA-Seq data demonstrating higher *ENTPD4* expression the tumor samples compared with normal samples (*P* = 0.01; n = 46). (**G**–**J**) IHC staining showing exclusive expression of ENTPD5 in the tumor. ENTPD5 was expressed at higher levels in node-positive cases, although the overall expression of the *ENTPD5* gene was similar in normal and tumor samples.

**Figure 6:**
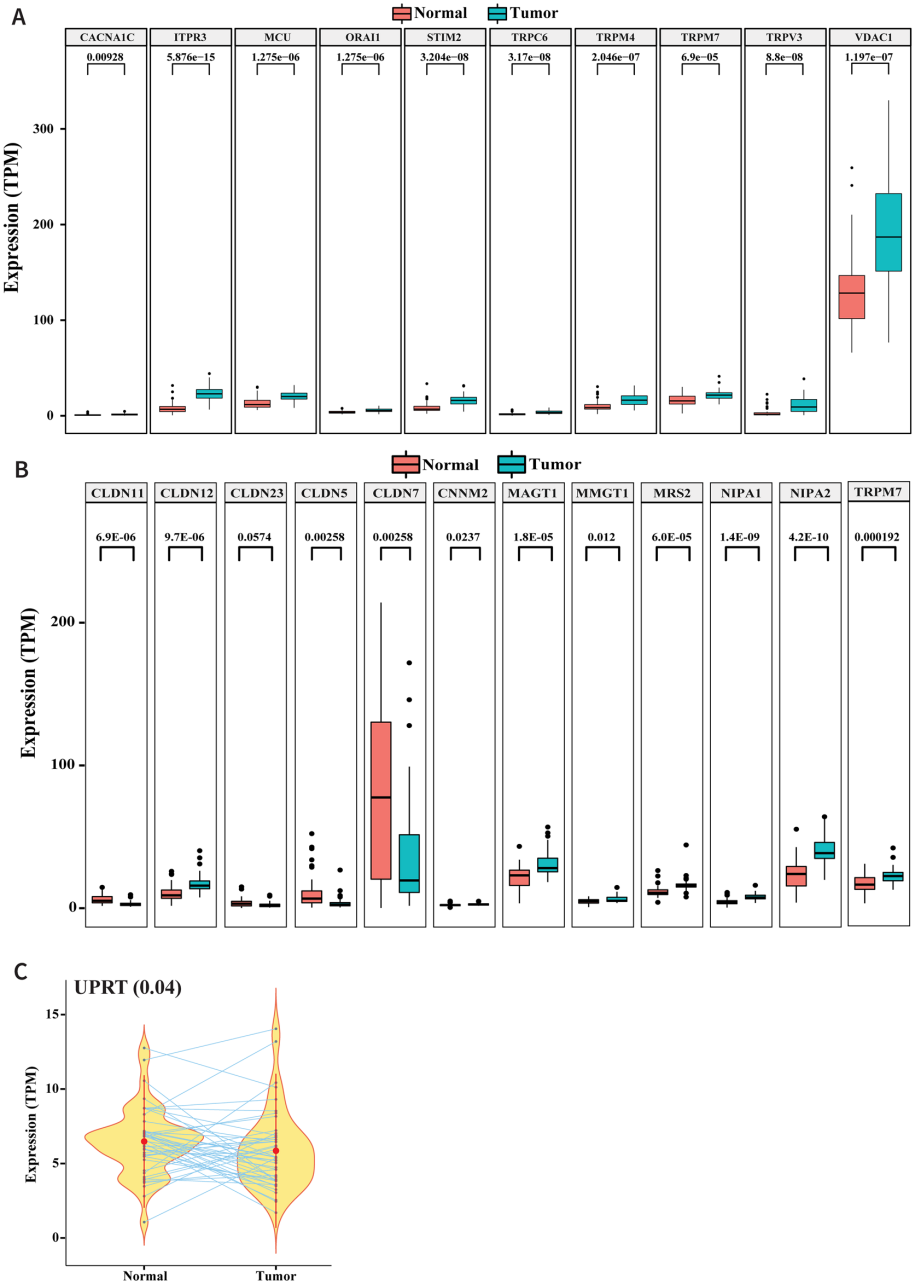
UMP to uracil production. (**A**) RNA-Seq data analysis showing Ca^2+^ and (**B**) Mg^2+^ signaling gene enrichment  (**C**) RNA-Seq data analysis showing significant upregulation of UPRT in tumor samples (*P* = 0.04; n = 46).

The P2Y6 receptor binds UDP preferentially, and its polar residues R103 and R287 interact with the phosphate and glyceryl moieties of the ligand ^21,22^. The endocytic pathway is known to promote receptor-mediated signaling responses. The arrest in isoforms bind to components of endocytic lattices (clathrin heavy chain, AP2 alpha subunit, and PIP2), promoting GPCR clustering in clathrin-coated pits. GPCRs that engage with the clathrin-dependent endocytic machinery are internalized and delivered to early endosomes ^23^. Although the P2Y6 receptor is normally located on the cell membrane, we also detected expression of the P2Y6 receptor in the cytosol (Fig. 5A). IHC showed that the P2Y6 receptor protein was localized in the cytoplasm of tumor cells at the invasive tumor edges where the cells were in close proximity to immune cells (Fig. 5A), indicating the possibility of UDP-bound endocytosis of the P2Y6 receptor. Other GPCR subtypes that are internalized by endosomes dissociate from the bound ligand(s) ^23^. IHC also revealed expression of the UDP-specific ENTPD4, with intense staining of the protein in a high percentage of immune cells in contrast to faint-to-moderate staining in tumor cells. In addition, tumor cells at the invasive edges showed moderate cytoplasmic staining (Fig. 5B,C). ENTPD4 expression was also significantly upregulated in node-negative cases (Fig. 5D). Although ENTPD4 was upregulated in immune cells, its expression in tumor cells appeared to be consistent, irrespective of the lymph node status (Fig. 5E). The genomic data showed higher expression of *ENTPD4* in tumor samples than in normal samples (Fig. 5F).On the other hand, ENTPD5 was not prominently expressed by immune cells, but was expressed at high levels in tumor cells. In addition, the higher expression of ENTPD5 correlated with lymph node metastasis (Fig. 5G–I). RNA-Seq analysis indicated steady expression of *ENTPD5*in both tumor and normal samples (Fig. 5J). ENTPD4/5 dephosphorylates endocytically-transported UDP to produce UMP in a reaction that requires a substantial and continuous supply of calcium and magnesium ions. The RNA-Seq data revealed enrichment of genes linked to Ca^2+^ and Mg^2+^signaling in the tumor samples (Fig. 6A,B). The conversion of UMP to uracil is a bidirectional reaction (RHEA database ID: 13,020) regulated by Mg^2+^/Ca^2+^ and uracil phosphoribosyltransferase (UPRT) ^24^. UPRT is required for the conversion of uracil to UMP; however, UPRT was not significantly upregulated in our cases (Fig. 6C), which accounts for the accumulation of uracil in the GB-OSCC tissues in this study.

## Discussion

In this study, we investigated the presence of key metabolites in oral cancer using proton NMR spectroscopy to identify metabolic biomarkers of GB-OSCC. We found that uracil was expressed in 83.09% of tumor tissues and pyrimidine metabolism was active in GB-OSCC; these results correlated well with IHC and RNA sequencing data. To the best of our knowledge, no previous study has demonstrated the presence of uracil as a metabolite in GB-OSCC tissues, indicating the potential value of uracil as a biomarker.

Due to its high reproducibility and capacity for quantitation, NMR has been used successfully to identify cancer biomarkers ^25,26^. Recently, Boguszewicz et al.^27^ successfully introduced NMR-based metabolic profiling in patients with head and neck squamous cell carcinoma. Metabolites and metabolic genes have also been identified using other spectroscopy methods applied to investigate radiation-induced changes in such patients. However, most relevant studies in this area were performed using saliva or serum samples ^27–29^, where the source of the metabolites could not be confirmed and the pathways were not delineated. In the present study, NMR analysis revealed significant differences in the spectral patterns of tumor samples compared to the normal samples, with the characteristic peaks for uracil detected at δ5.80 and δ7.54 in tumors (Fig. 1A). Previous metabolic profiling of gastric cancer using NMR ^30^ and a mass spectroscopy-based study on salivary metabolites in oral cancer identified uracil as one of the main metabolites ^31^, although the expression of uracil in oral cancer was not detected to be due to metabolic changes in tumor tissues. Although our tumor samples contained other co-metabolites that were upregulated and downregulated compared to normal tissues, we focused on uracil owing to its expression in 83.09% of tumors (Fig. 1C). Our NMR analysis of tumor tissues revealed significant changes in pyrimidine metabolism, of which uracil is an inherent component (Fig. 1B).

In the TME, cells are lysed by necrosis or apoptosis (Fig. 2A), thereby releasing intracellular molecules including metabolites. We detected upregulation of two pro-necrotic genes, *PGAM5* and *DFNA5*,in tumor samples compared to normal samples. Furthermore, *DFNA5*expression correlated with node-positive status, while *PGAM5*expression correlated with node-negative status (Fig. 2B–K). Necrosis is indicative of cell death, but due to the self-repair activity of tumor cells, the necrotic zone can be completely masked by tumor cells. Furthermore, necrosis may occur within a very small area or in a larger region within the stroma, which is often considered a poor prognostic factor. Our p53 mutation data supports the activation of the repair mechanism in the tumor samples (Supplementary Data 5). These findings are consistent with those of previous studies, in which pro-necrotic genes were shown to be upregulated in head and neck cancer as well as in other cancers ^32,33^. In human breast cancer, it has been shown that necrosis can occur through finely tuned regulation of a series of pro-necrotic genes including *MLKL, RIPK1, RIPK3, PGAM5,* and *DFNA5* ^20^. However, in the present study, only *PGAM5* and *DFNA5* were found to be relevant to necrosis in GB-OSCC.

Intracellular molecules, including metabolites, are released from necrotic cells into the TME, and utilized by high energy-demanding cancer cells. The main focus of the current study was to understand the pyrimidine metabolism pathway and the underlying mechanism of uracil formation. IHC and RNA-Seq data confirmed over expression of CD39 and CD73in cancer tissue, which supports the adenosine-based pathobiology of tumor cells (Fig. 3A–G) observed in other studies ^11,12,14^. An active CD39/CD73 pathway in purine metabolism is known to produce an immunosuppressive environment ^11,12^. In the CD39/CD73 pathway, large amounts of extracellular ATP produced in the hypoxic TME are degraded to adenosine via ADP and AMP by CD39 and CD73, respectively. As a substrate for CD39, UTP can also be converted to UDP in the TME. The abundant expression of CD39 in immune cells indicates the first hydrolysis event of UTP.

CD39 metabolizes extracellular ATP and ADP to AMP, therefore it is a key regulator of purinergic signaling in cancer. In a recent study, Vadlamani et al*.* showed that CD39 activity initially increases with the ATP/ADP concentration, but at higher ATP/ADP concentrations, there is a marked decrease in the activity of CD39. In contrast, such inhibition is not applicable to the pyrimidines UDP and UTP. Therefore, the CD39-mediated purinergic metabolism appears to be OFF at higher concentrations of ATP/ADP, while pyrimidine metabolism continues to be ON ^34^.

Although our transcriptomic data (Supplementary Data 5) and the p53 mutation status in our samples suggests that the extracellular pool of dUTP could have been neutralized by deoxyuridine triphosphate nucleotidohydrolase (dUTPase), aberrant p53 causes suppression of this enzyme ^35^. UDP from the TME enters the cell by binding to the P2Y6 receptor, which is normally present on the cell membrane. IHC-stained slides showed intense membrane and cytoplasmic expression of the P2Y6 receptor in tumor-infiltrating immune cells. Tumor cells close to the immune cells showed cytoplasmic granular positivity, while cells distant from the immune cells showed only membrane-positivity, providing evidence of P2Y6 receptor internalization by tumor cells (Figs. 3H, 5A). Inside the cell, ENTPD4 further hydrolyzes UDP to form UMP, which is then converted to uracil in a reaction that requires calcium and magnesium ions ^14^. The ENTPD4 staining pattern was similar to that of the P2Y6 receptor, with intense staining of immune cells as opposed to tumor cells, which exhibited cytoplasmic staining when in close proximity with immune cells (Fig. 5B–E). Therefore, these findings indicate that the UDP produced accumulated in and is utilized by the cancer cells.

The overexpression of CD39, CD73, P2Y6 receptor, and ENTPD4 proteins identified in tumor cells by IHC, was consistent with the over expression of their corresponding genes. ENTPD5 staining showed weak or no expression in immune cells, but was detected in tumor cells; however, the corresponding gene was not over expressed (Fig. 5G–J). Interestingly, there was concomitant upregulation of uracil, CD39, and CD73 in node-positive patients (Figs. 1E, 3D,G), indicating that the progression of GB-OSCC depends on increased uracil, CD39, and CD73 expression. Our results also show that CD39, CD73, and metabolic uracil are associated with lymph node metastasis and disease progression. Additionally, no characteristic uracil-specific peaks were detected in the NMR analysis of the GB-OSCC primary cell line, indicating that UDP is produced solely in the TME and subsequent synthesis of uracil occurs intracellularly. The expression of the pro-necrotic genes correlated with the lymph node status (node-positive and node-negative), signifying an active anti-necrotic mechanism that protects the tumor cells.

The impact of purine and pyrimidine metabolism on cancer progression has been reviewed ^30,36^. In some solid tumors (hepatocellular carcinoma and triple-negative breast cancer), catalytic degradation of pyrimidines promotes the mesenchymal-like state, leading to epithelial-to-mesenchymal transition ^37^. Dysregulated uracil expression associated with epigenetic alterations has been noted in hematologic malignancy, colon cancer, and prostate cancer ^36,38^. Like other nucleotidases, CD39 promotes tumor metastasis and is associated with a poor prognosis. Nevertheless, the therapeutic potential of pyrimidines in the TME has not been widely explored. A comprehensive understanding of the crosstalk between CD39 and pyrimidine metabolism, especially the role of enriched uracil in the prognosis of oral cancer, is crucial for the identification of strategies to improve the patient outcomes. To terms of the origin of uracil in oral cancer, we predicted that UDP was produced in the TME by the hydrolysis of UTP by CD39. CD39 enrichment in the stroma confirmed that the first hydrolysis event of UTP to UDP is mediated by the CD39 ectoenzyme (Fig. 3A). The increase in CD39 expression correlated with the presence of lymph node metastasis, supporting its role in the poor prognosis of patients with GB-OSCC (Fig. 3D). Tumor cells expressed P2Y6 receptor in the metabolically active regions of tumors at the invasive margins, which in turn promoted accumulation of the UDP produced in the nearby stroma. The UDP specificity of the P2Y6 receptor has been investigated widely in other cancer types ^39–41^; however, to the best of our knowledge, P2Y6 receptor functions and UDP-bound P2Y6 receptor endocytosis have not yet been explored in oral cancer. In this study, we noted higher expression of the *P2RY6* gene in tumor tissues (Fig. 3I), while IHC showed that the P2Y6 receptor was expressed both in the stroma and tumor cell membrane. However, cells at the invasive tumor margins showed over expression (Fig. 3H), indicating P2Y6 receptor expression dependency in the more energy-demanding cells as well as in the metabolically active regions. Interestingly, although the P2Y6 receptor is essentially a membrane protein, tumor cells at the invasive edges also expressed the P2Y6 receptor in the cytoplasm (Fig. 5A). This observation indicated that upon binding of extracellular UDP to the P2Y6 receptor, UDP is transported to the cytoplasm through endocytosis. The other UDP-specific, P2Y-subtype genes (*P2RY2* and *P2RY4*) were not significantly expressed and the UDP-glucose-specific *P2RY14* gene was downregulated (Fig. 4), suggesting that UDP is internalized solely via the P2Y6 receptor.

**Figure 7:**
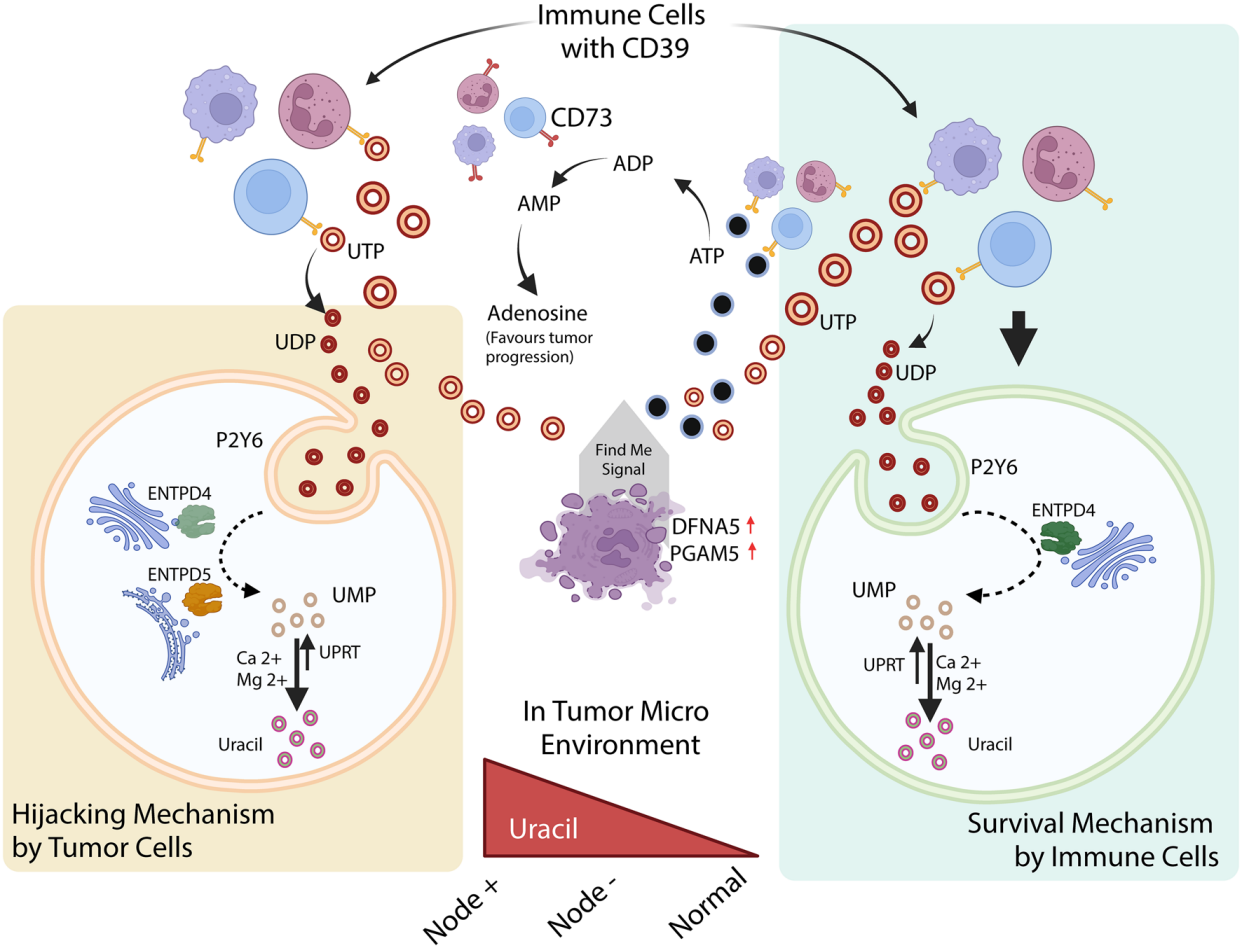
Schematic representation of the TME conditions for uracil production in GB-OSCC, in which the immune cells followed the survival mechanism and the tumor cells followed the hijacked mechanism.

*ENTPD4* encodes an endonuclease that has recently been reported to be active in gastric cancer ^42^. The encoded protein is localized mainly in the Golgi and lysosomes. The current study showed that ENTPD4 was over expressed in GB-OSCC tumor cells. On the other hand, expression of ENTPD5 endonuclease was found to correlate with the clinical stage and metastasis. In other cancer types, ENTPD5 knockdown resulted in the inhibition of cell proliferation, cell migration, and cell cycle arrest ^43^. Due to their UDP-substrate specificity, the intracellular endonucleases ENTPD4/5hydrolyze internalized UDP to UMP via a calcium- and magnesium-dependent pathway ^44^. Furthermore, ENTPD4/5 have been demonstrated to mediate mutant p53 gain-of-function activity in a host of cellular mechanisms, such as clonogenic growth, architectural tissue remodeling, migration, and invasion ^45^.In our cases, we detected the enrichment of intracellular calcium and magnesium signaling regulators (Fig. 6A,B).The STRING interaction network also supported the interaction of ENTPD4 and ENTPD5 activity (Supplementary Data 6). Therefore, our demonstration of the presence of uracil in the tumor samples indicates that this metabolite was generated from UMP in a bidirectional reaction that depends on Mg^2+^ and is catalyzed by uracil phosphoribosyltransferase (UPRT) ^23^. Downregulation of the *UPRT* gene restricts the conversion of uracil to UMP (Fig. 6C), whereas the upregulation of several Mg^2+^ signaling-related genes, including *CLDN12, MAGT1, NIPA1,* and *NIPA2,* supports the conversion of UMP to uracil (Fig. 4B).

## Conclusion

To summarize our findings, we used NMR to elucidate the over expression of uracil and the correlation of uracil expression with disease aggressiveness in GB-OSCC. Thus, our study highlights the importance of metabolic uracil not only as a prognostic marker in GB-OSCC, but also for screening and follow-up (in case of recurrence). Furthermore, the expression of additional IHC markers, such as CD39, CD73, P2Y6 receptor, and ENTPD4/5, and their spatial distribution patterns within the TME could be a signature for the disease progression. Based on our findings, we also ascertained the precise pathway(s) involved in uracil formation (Fig. 7), which could be useful in identifying potential therapeutic targets.

### Supplementary Information

Supplementary Information 1.

Supplementary Information 2.

Supplementary Information 3.

Supplementary Information 4.

Supplementary Information 5.

Supplementary Information 6.

## Data Availability

Metadata and Raw RNA_seq counts have been submitted and can be found under the bioproject ID PRJNA882808; GEO: GSE213862. However, if additional information is required, the corresponding author may be contacted.
