# Supplementary material for: Uracil as a biomarker for spatial pyrimidine metabolism in the development of gingivobuccal oral squamous cell carcinoma
Source: Sci Rep. 2024 May 21;14:11609. doi: 10.1038/s41598-024-62434-z (PMC11109148; doi:10.1038/s41598-024-62434-z)
Supplement: Supplementary file 1 — Supplementary Information 1. [file 41598_2024_62434_MOESM1_ESM.docx]

**Supplementary data 1.** Sample preparation from tissue sample for the NMR study.

**NMR sample preparation**

**Apparatus, Materials and Reagents:**

1. Microfuge tubes (1.5 ml & 2 ml) and Microfuge Rack.

2. Cooling centrifuge compatible to microfuge tubes.

3. Disposable polypropylene pestle for 1.5 ml microfuge tube.

4. Vortex machine.

5. SpeedVac/Lipolyzer.

6.p^H^ meter.

7. 0.22 syringe filter.

8. Micropipette (20-200 ul, 100-1000 ul, with compatible tips).

9. HPLC-grade methanol (*to make 80% (v/v), add 10 ml of water to 40 ml MeOH in polypropylene measuring tube*).

10. LC/MS-grade water.

11. PBS tablets/Na_2_HPO_4_.2H_2_O, KH_2_PO_4_, NaCl, KCl.

12. D_2_O.

13. NaN3.

**14. 5 mm NMR tube**

**15. NaOH**

**16. HCl**

***Task 1: Protocol for metabolites extraction from tumors tissues (start with 10–15 mg of solid tissue)***

**Step 1:**Add 500 μl of 80% (vol/vol) HPLC-grade methanol

(cooled to − 80 °C) to fresh or frozen tissue piece(s) in a

1.5-ml or 2.0-ml microcentrifuge tube.

**↓**

**Step 2:**Smash/grind for 1–2 min with small pestle/tissue grinder

on dry ice in the tube, vortex for 1 min at 4–8 °C and

incubate for 4 h at − 80 °C.

**↓**

**Step 3:**Centrifuge at 14,000g (or the highest speed) for 10 min

using a refrigerated centrifuge (4–8 °C).

**↓**

**Step 4:**Transfer the supernatant to a new 1.5-ml microcentrifuge

tube; store at − 80°C until Step (8).

**↓**

**Step 5:**Add 400 μl of 80% (vol/vol) methanol (cooled to − 80

°C) to the precipitate.

**↓**

**Step 6:**Vortex for 1 min at 4–8 °C, and then incubate for 30 min

at − 80 °C.

**↓**

**Step 7:**Centrifuge at 14,000g for 10 min (4–8 °C).

**↓**

**Step 8:**Transfer and combine the supernatant from bothextractions.

**↓**

**Step 9:**Centrifuge again at 14,000g for 10 min (4–8 °C).

**↓**

**Step 10:**Transfer the supernatant to a new 1.5-ml microcentrifugetube.

**↓**

**Step 11:**SpeedVac/lipolyzed to a pellet using no heat.

(This dried metabolite samples can bestored at − 80 °C for several weeks.)

**Task 2: Reconstitution of Dried metabolites for NMR spectroscopy**

Sample reconstituted in 1 ml phosphate buffer (PBS) prepared (0.1 M) in D2O (pH=7.4), containing NaN3 (prevent bacterial contamination and stabilize glutathione).

**↓**

Homogenized the sample and adjusted at pH=7.40 +/−0.05,

**↓**

Filtered through 0.22 um filter,

**↓**

And then analyzed using a 5-mm NMR tube.
