# Supplementary material for: Uracil as a biomarker for spatial pyrimidine metabolism in the development of gingivobuccal oral squamous cell carcinoma
Source: Sci Rep. 2024 May 21;14:11609. doi: 10.1038/s41598-024-62434-z (PMC11109148; doi:10.1038/s41598-024-62434-z)
Supplement: Supplementary file 2 — Supplementary Information 2. [file 41598_2024_62434_MOESM2_ESM.docx]

**Supplementary data 2.**Antibody validation method of monoclonal anti-CD39, CD73, P2Y6 receptor, and ENTPD5) and polyclonal anti-ENTPD4 antibodies.

Four out of the five antibodies are recombinant rabbit monoclonal antibodies from Abcam (anti-CD39, CD73, P2Y6 receptor, ENTPD5) and one is a polyclonal from Sigma-Aldrich (anti-ENTPD4). Both Companies are reputed and their antibodies are highly characterized and extensively validated and confirmed for specificity.

On receiving the antibodies in cold chain, we have validated and standardized them in our lab as follows:

Validation was done as per the manufacturers guidelines, on the Leica BOND automated platform, with special attention given to a) maintaining the pH for antigen retrieval b) Using multiple dilutions of the primary antibody c) Using both positive and negative controls.

The secondary antibody is supplied as a kit by Leica called BOND Polymer Refine Detection Kit where the reagents are ready to use. The reagents in this kit have a) Secondary antibody b) Peroxidase blocking reagent c) Polymer reagent d) DAB chromogen e) Haematoxylin counterstain.
