# Supplementary material for: Uracil as a biomarker for spatial pyrimidine metabolism in the development of gingivobuccal oral squamous cell carcinoma
Source: Sci Rep. 2024 May 21;14:11609. doi: 10.1038/s41598-024-62434-z (PMC11109148; doi:10.1038/s41598-024-62434-z)
Supplement: Supplementary file 3 — Supplementary Information 3. [file 41598_2024_62434_MOESM3_ESM.docx]

**Supplementary data 3***.* Digitization of the IHC slides and Image processing & analysis.

**Microcopy Details**

The IHC-based scoring methods were designed in QuPath software to segment cells based on staining intensity. The immune markers at the tumor invasive margins (IM) and tumor centre (CT) were analyzed.

**Digitization of the IHC slides:** The tissue microarray images were scanned using an automated bright field microscope(Aperio Versa 8, Leica, Germany) under 20x plan objective (NA 0.55, with finalmagnification × 100) with a resolution of 0.275 μm per pixel. The images were digitized to apixel range of 1388 × 1040 pixels using CMOS camera. The image grabbing andpreprocessing software package was inbuilt into the support of AperioImageScope(v12.3.3.5048) software platform.

**Image processing & analysis:** The selected images were annotated with disease grading, staging and otherclinicopathological parameters by the expert oncopathologists. The resolution of the imageswas 2048 X 2048 with pixel size of 5.5 µm. The images were extracted and converted into (.tiff) filefrom (.scn) extension file for further image analysis. Image processing and analysis wasperformed using Qupath (0.1.2). Selected .tiff files were opened in QuPath andfollowing work flow followed:

1. Set image type: Brightfield (H-DAB) was selected

2. Set color deconvolution stain: Stain 1= Hematoxylin; Stain 2= DAB

3. Positive cell detection: In this step various parameters were defined for cell segmentation and quantification.

Table 1

| Parameter | Value |
| --- | --- |
| Detection image brightfield | Optical density (OD) sum |
| Requested pixel size microns | 0.5 |
| Background radius microns | 8.0 |
| Median radius microns | 0.0 |
| Sigma microns | 1.5 |
| Min area microns | 10.0 |
| Max area microns | 400 |
| Threshold | 0.1 |
| Max background | 2.0 |
| Watershed post process | true |
| Exclude DAB | false |
| Cell expansion microns | 5.0 |
| Include nuclei | true |
| Smooth boundaries | true |
| Make measurement | true |
| Threshold compartment | Cell: DAB OD mean |
| Threshold positive 1 | 1.5 |
| Threshold positive 2 | 0.1878/0.4197/1.5 |
| Threshold positive 3 | 0.2201/0.2197/0.4197 |
| Single threshold | False |
| True= selected, False= not selected | |

Immuno-scoring for both IM and CT were performed for each selected biomarker using QuPath software. The percentage values of DAB positive cells out of hematoxylin positive cells were recorded. Tumour center (CT) area of ~500 µm distance from invasive margin (IM) was selected for scoring immune cells using brush tool and wand tool wherever applicable. To generate the percentage positive scores of the individual markers, thresholds were developed in Qupath software (as per Table 1).
