# Supplementary figures and images for: Uracil as a biomarker for spatial pyrimidine metabolism in the development of gingivobuccal oral squamous cell carcinoma
Source: Sci Rep. 2024 May 21;14:11609. doi: 10.1038/s41598-024-62434-z (PMC11109148; doi:10.1038/s41598-024-62434-z)

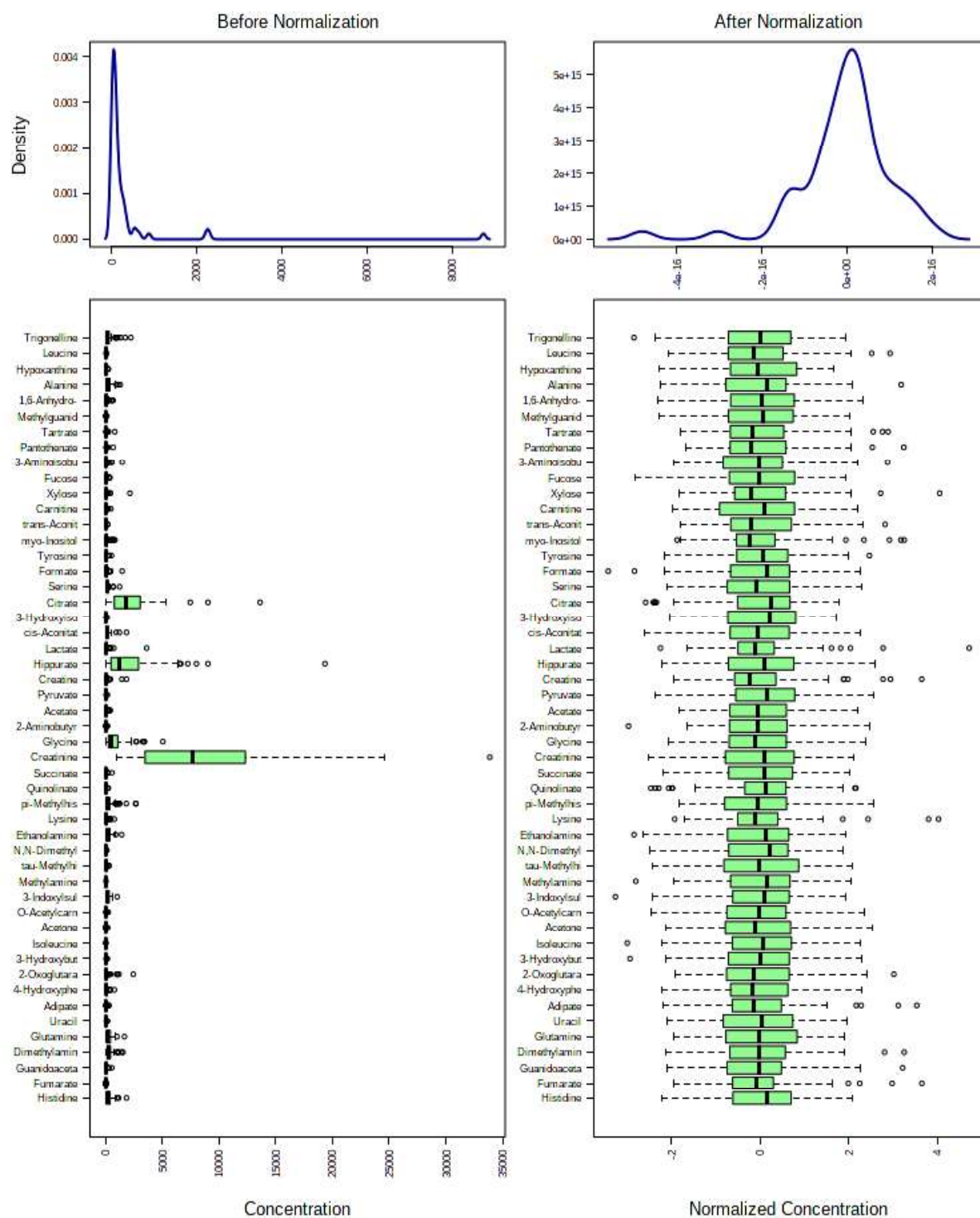

Supplementary data 4. Data normalization for NMR data analysis.

Supplement: Supplementary file 4 — Supplementary Information 4. [file 41598_2024_62434_MOESM4_ESM.pdf]

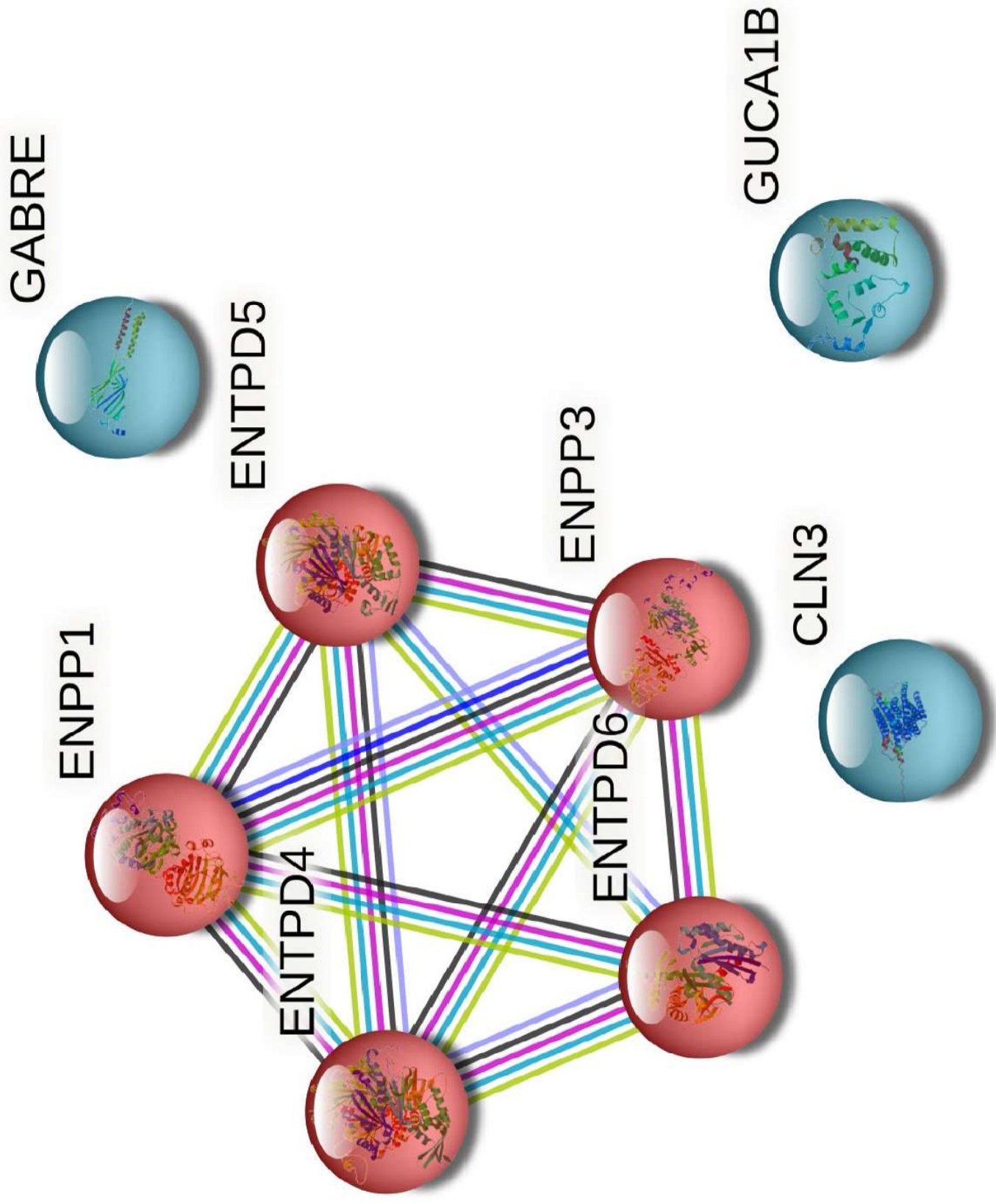

Supplement: Supplementary file 6 — Supplementary Information 6. [file 41598_2024_62434_MOESM6_ESM.pdf]
